# Tibial nerve compression due to osteochondroma of the fibular head: A case report

**DOI:** 10.1097/MD.0000000000036059

**Published:** 2023-11-10

**Authors:** Young-Keun Lee, Ji Woong Ho

**Affiliations:** a Department of Orthopedic Surgery, Research Institute of Clinical Medicine of Jeonbuk National University – Biomedical Research Institute of Jeonbuk National University Hospital, Jeonju, Jeonbuk, Republic of Korea.

**Keywords:** fibular head, osteochondroma, tibial nerve, tibial nerve palsy

## Abstract

**Rationale:**

Osteochondroma is one of the most common primary benign bone tumors. In most cases, this disease is asymptomatic. However, it may become symptomatic owing to nerve and vascular compression when it affects the knee joint. Isolated tibial nerve palsy caused by proximal fibular osteochondroma is rare.

**Patients concerns:**

A 60-year-old male, was treated for degenerative arthritis of the right knee, referred to the right great toe flexion limitation that occurred 3 weeks prior.

**Diagnoses:**

Magnetic resonance imaging revealed compression of the tibial nerve and surrounding muscles due to an osseous lesion in the fibular head. A nerve conduction test confirmed tibial neuropathy in the right lower leg.

**Interventions:**

Exploratory surgery was performed to decompress the tibial nerve and remove the bony lesion histopathologically diagnosed as an osteochondroma.

**Outcomes:**

Fifty-five months postoperatively, toe flexion recovered to normal. No recurrence of osteochondroma was observed.

**Lessons:**

As in our case, if a bony lesion is diagnosed on radiographs with neurological symptoms, early decompression surgery is necessary. Moreover, since it can be misdiagnosed as a simple bony spur, magnetic resonance imaging and tissue biopsy are also indicated.

## 1. Introduction

Tibial nerve is the large terminal branch of the 2 main muscular branches of the sciatic nerve. The tibial nerve innervates to the muscles of the lower leg and foot, specifically the gastrocnemius, soleus, plantaris, tibialis posterior, flexor digitorum longus, and flexor hallucis longus. The tibial nerve has cutaneous branches that supply sensation to the skin in an area from the outside of the knee down the back of the calf to the outside portion of the foot and most of the sole of the foot.^[1]^

Osteochondroma is the most common form of primary benign bone tumors. It is usually found in the second and third decades of life.^[2]^ The vast majority of these tumors present as solitary, nonhereditary lesions. Solitary osteochondromas show a predilection for the metaphysis of the long tubular bones, particularly the femur (30%), humerus (26%), and tibia (43%). Lesions affecting the fibula, carpal, and tarsal bones, patella, and spine are rare.^[3,4]^

Several cases of neuropathy arising from tibial nerve compression affecting the knee joint, either due to a Baker cyst or compression beneath the tendinous arch at the origin of the soleus muscle, have been reported.^[5,6]^ However, there are no cases of isolated tibial nerve compression due to osteochondroma, other than the 1 reported by Gökkuş et al^[7]^ Herein, we report a case of neuropathy due to tibial nerve compression due to osteochondroma of the fibular head, a rare location for the tumor, occurring in an uncommon age group. Herein, we report the present case with a literature review.

### 1.1. Consent

The patient signed an informed consent form for the publication of this case report and any accompanying images. The ethical approval for this study was waived by the ethics committee of Jeonbuk National University Hospital because it was a case report with fewer than 3 patients (2021-12-044).

## 2. Case presentation

**Figure 1.**
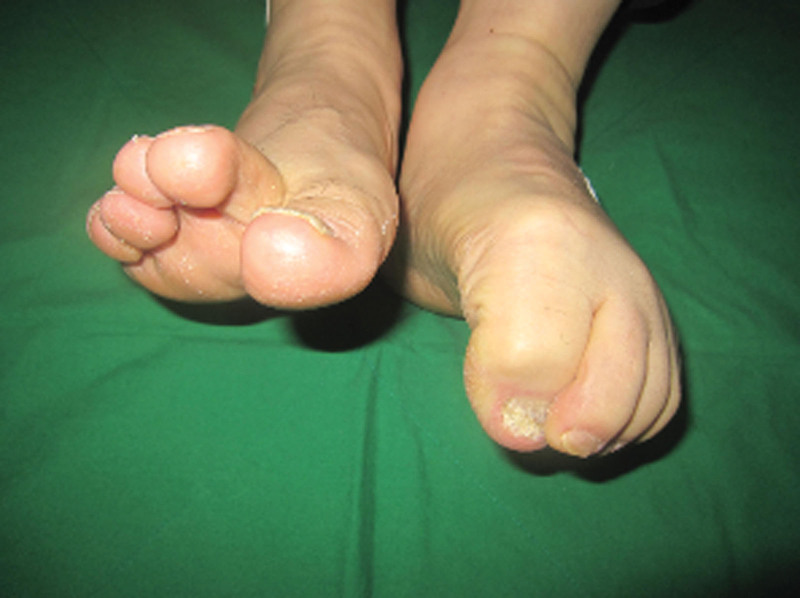
Preoperative physical examination showing limited flexion in right toes, FHL and FDL. FHL = flexor halluscis longus, FDL = flexor digitorum longus.

**Figure 2.**
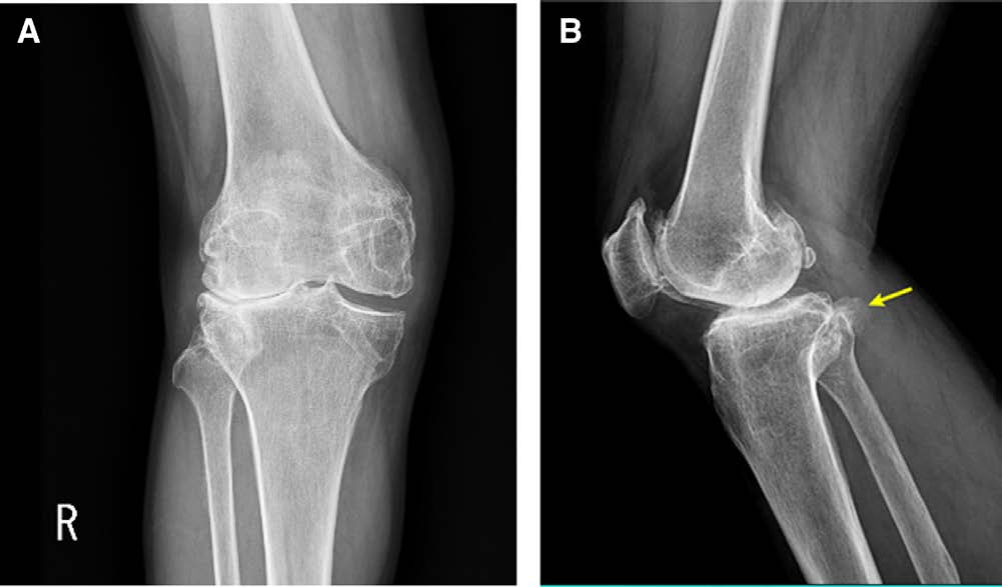
(A and B) Preoperative radiographs of the right knee, showing osseous lesion protruding posteriorly to the fibular head (arrow), bone spurs in the distal femur and proximal tibiofibular, and K–L grade 4 OA with joint space narrowing. K–L grade = = Kellgren–Lawrence grade, OA = osteoarthritis.

A 60-year-old male was admitted with chief complaints of flexion limitation in the right 4 toes, including the great toe that began 3 weeks ago, with a tingling sensation in the right great toe, 4, 5 toes, plantar area, and heel that began 1 month prior to presentation. The patient underwent post-traumatic reconstruction of the anterior and posterior cruciate ligaments of the right knee in 1984. On physical examination, hypesthesia in the distribution of the right tibial nerve, along with toe flexion limitation, was observed (Fig. 1). No direct tenderness or swelling was observed on the legs. Blood circulation was normal. The American orthopedic Foot and Ankle Society scores were 54.^[8]^ Visual score for pain was 8. Plain radiological findings showed Kellgren-Lawrence grade 4 osteoarthritis (OA)^[9]^ accompanied by bony spurs in the distal femur, patella, and proximal tibiofibula, along with joint space narrowing, especially affecting the lateral side of the right knee (Fig. 2).

**Figure 3.**
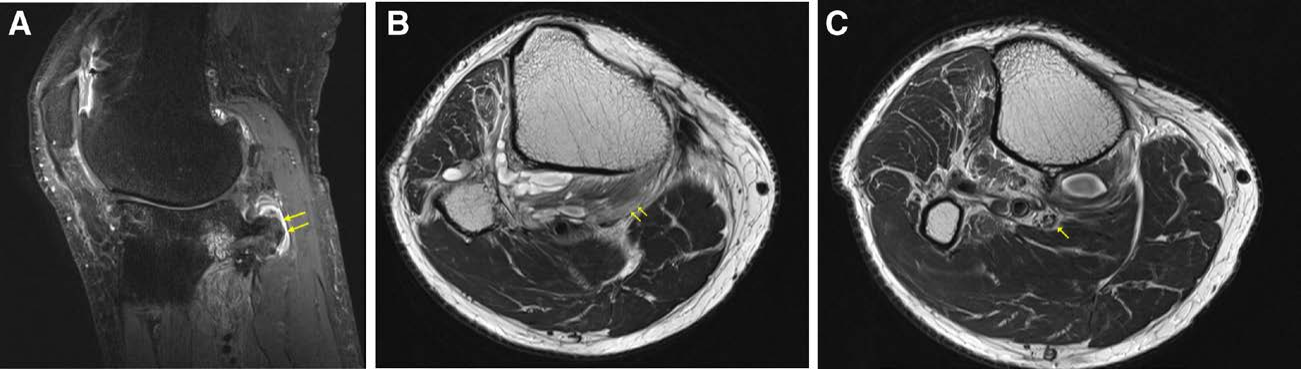
(A–C) T2 weighted images. (A) Sagittal view showing osseous lesion associated with the medullary cavity of the fibula (arrows). (B, C) Axial image showing popliteus muscle edema (arrows) and tibial nerve swelling (arrow) around the knee joint.

Magnetic resonance imaging showed severe degenerative changes inside the joint and surrounding loose bodies, with bony spurs in the fibular head connected to the medullary canal. Neuropathy was suspected because of swelling of the popliteal area, as well as edema of the tibial nerve and surrounding muscles (Fig. 3A–C).

Electromyography and nerve conduction velocities revealded tibial nerve neuropathy between the tibialis posterior and flexor digitorum longus muscles. Accordingly, an exploration was planned.

**Figure 4.**
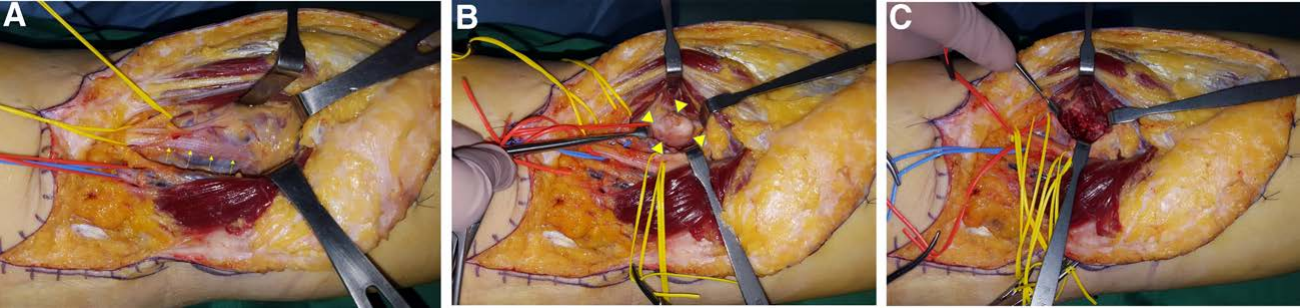
(A–C) Intraoperative photographs showing (A) compression of the tibial nerve (arrows) caused by a posteromedially protruding osseous lesion and (B) tibial nerve compressed between the lateral head of gastrocnemius muscle due to an osseous lesion (arrow heads). (C) Decompression of the tibial nerve was confirmed.

**Figure 5.**
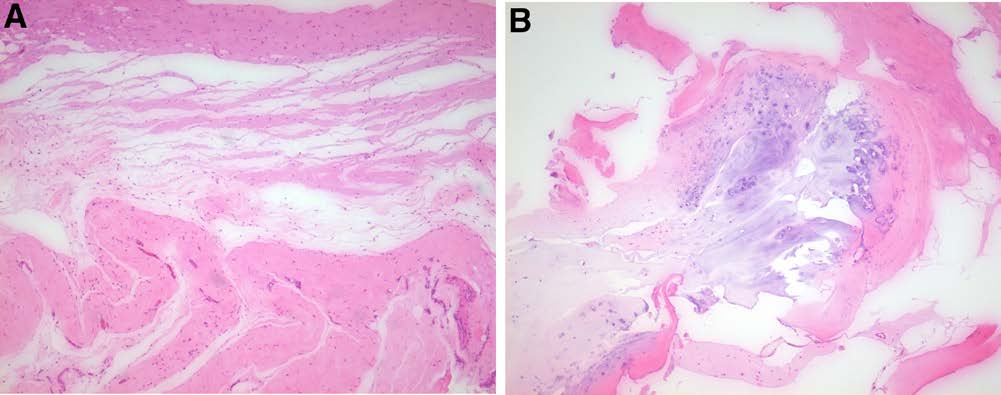
(A and B) Histopathologic findings. (A) Cystic change of bony lesion, (B) Osteochondroma without malignancy changes (Hematoxylin-eosin, original magnification x100).

A zigzag incision was made in the popliteal area under general anesthesia with the patient in a prone position. The hamstring muscles were carefully detached to examine the tibial nerve, and popliteal artery, and veins. Intraoperative findings showed that the tibial nerve was compressed between a bone-like mass suggestive of osteochondroma or an osteophyte arising posteromedially in the fibular head. It also revealed a cystic soft tissue mass located above the bone mass, containing synovial fluid, a loss body, and the lateral gastrocnemius muscle (Fig. 4A). After careful detachment and examination of the tibial nerve, it was found that it was compressed between the lateral head of the gastrocnemius muscle due to a bony lesion (3 × 3 cm in size) developed behind the fibular head and a cystic lesion containing loose bodies and joint fluid (Fig. 4B). An excisional biopsy of the bony lesion followed by histopathological examination confirmed tibial nerve decompression (Fig. 4C). Postoperative imaging confirmed that the osseous lesion was sufficiently removed. Bony and soft tissue lesions were finally diagnosed as osteochondroma and ganglion, respectively, by histopathological examination (Fig. 5).

**Figure 6.**
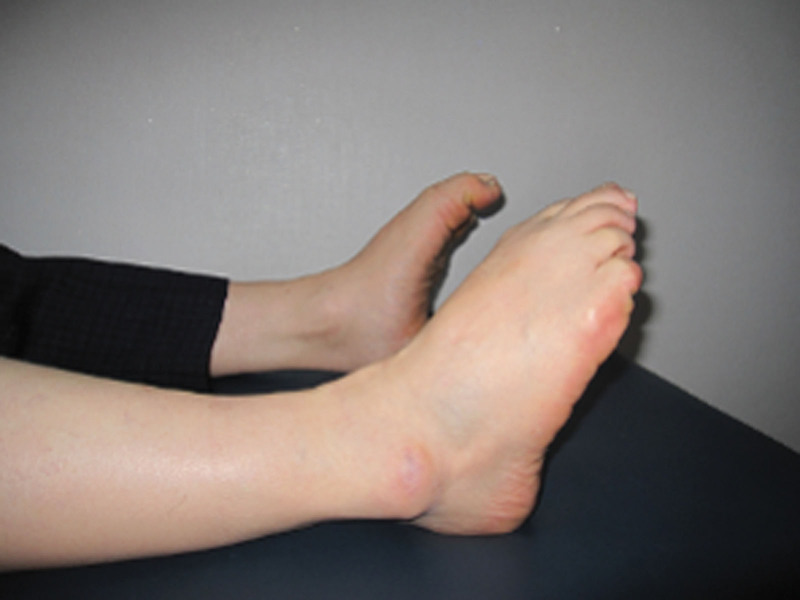
Photographs obtained at 55 months after operation showing excellent toe flexion.

**Figure 7.**
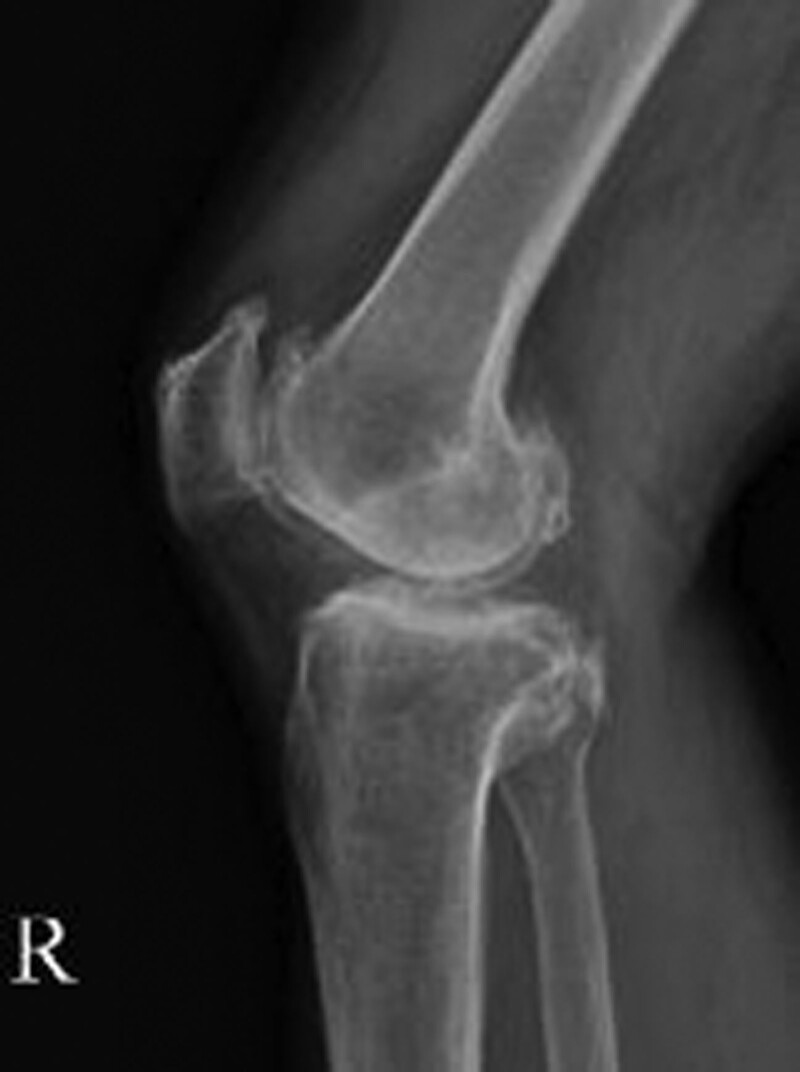
Postoperative follow-up plain radiographs at after 55 months after operation showing no recurrence of osteochondroma.

Fifty-five months after the surgery, his muscle strength recovered to a normal range of flexion. Except for intermittent plantar tingling, the patient did not complain of any foot pain (Fig. 6). Plain radiographs showed no recurrence of the fibular head (Fig. 7).

## 3. Discussion

The tibial nerve branches from the sciatic nerve at the superior angle of the popliteal fossa and continues its course down the center of the popliteal fossa to run alongside the posterior tibial artery between leg muscles. It passes posterior to the medial malleolus, where it branches into medial and lateral plantar nerves.^[1]^

Tibial nerve entrapment is rare compared with peroneal nerve entrapment because of the abundance of fat and muscle layers surrounding the tibial nerve. Nerve entrapment syndrome may occur along the course of the tibial nerve. They most commonly occur in the tarsal tunnel. Nerve entrapment near the knee joint is rare. Cases of tibial nerve and vascular compression that have been reported mainly include those caused by Baker cysts or nerve compression under the origin of the tendinous arch of the soleus muscle.^[5,6]^

Entrapment neuropathy around the knee joint may occur for various reasons, including direct injury, compressive injury, fibular fracture, ischemic neuropathy, spontaneous hematoma, tibiofibular joint cysts, and tumors.^[2,10]^ In particular, entrapment neuropathy of the fibular head is most frequently associated with the peroneal nerve because of its anatomic characteristics.

In the present case, the patient presented with impaired toe flexion and hypesthesia in an area innervated by the right tibial nerve. Based on this finding, entrapment neuropathy of the tibial nerve was suspected. In addition, preoperative radiological findings revealed multiple bone spurs in the distal femur, proximal tibia, and fibular head due to Kellgren–Lawrence grade 4 OA. Considering the patient’s age and plain radiographs, it was originally believed that the patient’s symptoms might be attributed to the presence of bony spurs due to severe OA. Therefore, surgeons should be cautious when treating patients with multiple bone spurs due to OA.

Osteochondroma is a common type of bone tumor that accounts for 30% of benign bone tumors and 10% to15% of all bone tumors.^[4]^ It is usually present in the epiphyses of the long bones. It is often found around 10 to 25 years of age. As most cases are asymptomatic, They are often detected incidentally on radiographs. It is commonly found around the knees. Its incidence is 40% in the distal femur near the knee and 15% to 20% in the proximal tibiofibular region.^[11]^ Abdel et al^[12]^ analyzed 121 cases of tumors in the proximal fibular region and found that osteochondroma was the most common (38%). Peroneal nerve symptoms occurred in 12% of cases, whereas tibial nerve symptoms were absent. Osteochondromas are space-occupying lesions that tend to grow outward from the center. However, in the present case, the osteochondroma grew inward from the posterior fibular head, compressing the tibial nerve. In addition, loss of normal alignment after severe trauma to the lateral knee compartment and the consequent knee valgus deformity in the standing position might have aggravated the compression of the tibial nerve by osteochondroma arising in the fibular head.

Complete surgical neurolysis is a critical treatment modality for tibial nerve compression caused by space-occupying lesions.^[13]^ On the contrary, most cases associated with isolated osteochondroma are treated using conservative methods with periodic monitoring.^[3]^ However, surgical excision is performed when the tumor size presents an aesthetic problem, if there is a pathological fracture, if symptoms of nerve or vascular compression appear, if there is limitation in the movement of joints, and if exacerbation of the tumor is suspected. If one of the indications for surgery is present, surgical intervention should not be delayed. Hence, in the present case, pain relief and improvement of sensory and motor functions were achieved by surgical intervention.

Osteochondroma of the fibular head is often accompanied by neurological symptoms in the legs owing to its anatomical significance. If radiographic findings confirm an osseous lesion and the patient shows clear signs of nerve-related symptoms, early decompression is necessary.

## 4. Conclusion

Tibial nerve neuropathy due to an osteochondroma of the fibular head adjacent to the knee joint is extremely rare. The present case was thought to have resulted from a change in knee joint alignment due to OA after severe trauma. Although rare, the diagnosis of tibial nerve compression can lead to rapid surgical treatment with good outcomes. Special care must be taken when diagnosing bony spurs due to OA around the knee joint.

## Author contributions

**Conceptualization:** Young-Keun Lee.

**Data curation:** Young-Keun Lee, Ji Woong Ho.

**Formal analysis:** Young-Keun Lee, Ji Woong Ho.

**Investigation:** Young-Keun Lee.

**Methodology:** Young-Keun Lee.

**Resources:** Young-Keun Lee.

**Software:** Young-Keun Lee.

**Supervision:** Young-Keun Lee.

**Validation:** Young-Keun Lee.

**Visualization:** Young-Keun Lee, Ji Woong Ho.

**Writing – original draft:** Young-Keun Lee, Ji Woong Ho.

**Writing – review & editing:** Young-Keun Lee, Ji Woong Ho.
